# Supplementary material for: Nutrient restriction enhances the proliferative potential of cells lacking the tumor suppressor PTEN in mitotic tissues
Source: eLife. 2013 Jul 9;2:e00380. doi: 10.7554/eLife.00380 (PMC3707060; doi:10.7554/eLife.00380)
Supplement: Supplementary file 1. — DOI: http://dx.doi.org/10.7554/eLife.00380.042 [file elife00380s007.docx]

**Supplementary file 1**

**Genotypes of experimental animals**

**Figure 1.** (A, B) *y, w*; (C, D) *y, w* / *y, w*; *PKB^3^*/*PKB^3^* / *y, w*; *PTEN^117^*/*PTEN^100^*; (E, F) *y, w* / *y, w*; *PTEN^117^*/*PTEN^100^*

**Figure 2.** (B, C) *y*, *w, hsFlp*/*y*, *w*; *FRT40 ubiGFP*/*FRT40 PTEN^117^* / *y*, *w, hsFlp*/*y*, *w*; *FRT40 ubiGFP*/*FRT40iso*; (D) *y*, *w, eyFlp*/*y*, *w*; *FRT40 ubiGFP*/*FRT40 PTEN^117^* / *y*, *w, eyFlp*/*y*, *w*; *FRT40 ubiGFP*/*FRT40iso*; (E) *y*, *w, eyFlp*/*y*, *w*; *FRT40 w^+^ cl*/*FRT40 PTEN^117^* / *y*, *w, eyFlp*/*y*, *w*; *FRT40 w^+^ cl*/*FRT40iso*

**Figure 3.** (A) *y*, *w, hsFlp*/*y*, *w*; *FRT40 ubiGFP*/*FRT40 PTEN^117^*; (B) *y*, *w, hsFlp*/*y*, *w*; *FRT40 ubiGFP*/*FRT40 PTEN^117^*/ *y*, *w, hsFlp*/*y*, *w*; *FRT40 ubiGFP*/*FRT40iso*; (C) *y, w, hsFlp, UAS-GFP*; *FRT40 tubGal80*/*FRT40iso*; *tubGal4*/+ / *y, w, hsFlp, UAS-GFP*; *FRT40 tubGal80*/*FRT40iso*; *tubGal4*/UAS-p35 / *y, w, hsFlp*, *UAS-GFP*; *FRT40 tubGal80*/*FRT40 PTEN^117^*; *tubGal4*/*+* / *y, w, hsFlp*, *UAS-GFP*; *FRT40 tubGal80*/*FRT40 PTEN^117^*; *tubGal4*/*UAS-p35*; (D) *y, w, hsFlp; FRT40 ubiGFP*/*FRT40 PTEN^117^*; *DE-Gal4*/+ / *y, w, hsFlp; FRT40 ubiGFP*/*FRT40 PTEN^117^*; *DE-Gal4*/*UAS-p35*; (E) *y*, *w, hsFlp*/*y*, *w*; *FRT40 ubiGFP*/*FRT40iso* / *y*, *w, hsFlp*/*y*, *w*; *FRT40 ubiGFP*/*FRT40* bsk^1^ / *y*, *w, hsFlp*/*y*, *w*; *FRT40 ubiGFP*/*FRT40 PTEN^117^* / *y*, *w, hsFlp*/*y*, *w*; *FRT40 ubiGFP, PTEN^117^*/*FRT40 bsk^1^*

**Figure 4.** (A) *y*, *w, hsFlp*/*y*, *w*; *FRT40 armLacZ*/*FRT40 PTEN^117^*; *tGPH;* (B) *y*, *w, hsFlp*/*y*, *w*; *FRT40 ubiGFP*/*FRT40 PTEN^117^* / *y*, *w, hsFlp*/*y*, *w*; *FRT40 ubiGFP*/*FRT40iso*; (C) *y*, *w*, *eyFlp*/*y*, *w*; *FRT82 ubiGFP*/*FRT82iso* / *y*, *w*, *eyFlp*/*y*, *w*; *FRT40 PTEN^117^*; *FRT82 PTEN^genomic rescue^*, *ubiGFP*/*FRT82iso* / *y*, *w*, *eyFlp*/*y*, *w*; *FRT82 ubiGFP*/*FRT82 PKB^3^* / *y*, *w*, *eyFlp*/*y*, *w*; *FRT40 PTEN^117^*; *FRT82 PTEN^genomic rescue^*, *ubiGFP*/*FRT82 PKB^3^* / *y*, *w*, *eyFlp*/*y*, *w*; *FRT82 ubiGFP*/*FRT82 PKB^1^* / *y*, *w*, *eyFlp*/*y*, *w*; *FRT40 PTEN^117^*; *FRT82 PTEN^genomic rescue^*, *ubiGFP*/*FRT82 PKB^1^*; (D) *y*, *w*; *GMR-Gal4*/+; *UAS-lacZ* / *y*, *w*; *GMR-Gal4*/*UAS-PTEN^RNAi^* / *y*, *w*; *GMR-Gal4*, *Thor1*/+; *EP(dFoxO)*/+ / *y*, *w*; *GMR-Gal4, Thor1*/*UAS- PTEN^RNAi^*; *EP(dFoxO)*; (E) *y*, *w*, *hsFlp*/*w*; *UAS-cherry-dFoxO*/+; *Act>CD2>Gal4*, *UAS-GFP*/+ / *y, w, hsFlp*/*w*; *UAS-cherry-dFoxO*/*UAS-PTEN^RNAi^*; *Act>CD2>Gal-4, UAS-GFP*/+; (F) *y, w, hsFlp, UAS-myrGFP*/*y, w*; *FRT40 tub-Gal80*/*FRT40 PTEN^117^*; *tub-Gal4*/+ / *y, w, hsFlp, UAS-myrGFP/y*, *w*; *FRT40 tub-Gal80*/*FRT40 PTEN^117^*; *tub-Gal4*/*UAS-Tsc1 UAS-Tsc2* / *y, w, hsFlp, UAS-myrGFPIy, w*; *FRT40 tub-Gal80*/*FRT40iso*; *tub-Gal4*/*UAS-Tsc1 UAS-Tsc2*; (G) *y, w, eyFlp*/*y, w*; *FRT40 tub-Gal80*/*FRT40iso*; *tub-Gal4*/+ / *y, w, eyFlp*/*y, w*; *FRT40 tub-Gal80*/*FRT40iso*; *tub-Gal4*/*UAS-Tsc1 UAS-Tsc2* / *y, w, eyFlp*/*y, w*; *FRT40 tub-Gal80*/*FRT40 PTEN^117^*; *tub-Gal4*/+ / *y, w, eyFlp*/*y, w*; *FRT40 tub-Gal80*/*FRT40 PTEN^117^*; *tub-Gal4*/*UAS-Tsc1 UAS-Tsc2*

**Figure 5.** (A, B) *y, w, hsFlp, UAS-myrGFP*/*y, w*; *FRT40 tub-Gal80*/*FRT40iso*; *tub-Gal4*/+ / *y, w, hsFlp, UAS-myrGFP*/*y, w*; *FRT40tub-Gal80*/*FRT40iso*; *tub-Gal4/UAS-Slif^anti^* / *y, w, hsFlp, UAS-myrGF*P/*y, w*; *FRT40 tub-Gal80*/*FRT40 PTEN^117^*; *tub-Gal4*/+ / *y, w, hsFlp, UAS-myrGFP*/*y, w*; *FRT40 tub-Gal80*/*FRT40 PTEN^117^*; *tub-Gal4*/*UAS-Slif^anti^*; (C) *y, w, hsFlp, UAS-myrGFP*/*y, w*; *FRT40 tub-Gal80*/*FRT40 PTEN^117^*; *tub-Gal4*/+ / *y, w, hsFlp, UAS-myrGFP*/*y, w*; *FRT40 tub-Gal80*/*FRT40 PTEN^117^*; *tub-Gal4/ UAS-Slif^anti^*; (D) *y, w, hsFlp, UAS-myrGFP*/*y, w*; *FRT40 tub-Gal80*/*FRT40iso*; *tub-Gal4*/+ / *y, w, hsFlp, UAS-myrGFP*/*y, w*; *FRT40 tub-Gal80*/*FRT40 PTEN^117^*; *tub-Gal4*/+ / *y, w, hsFlp, UAS-myrGFP*/*y, w*; *FRT40 tub-Gal80*/*FRT40 PTEN^117^*; *tub-Gal4/UAS-Slif^anti^*

**Figure 6.** (A) *y*, *w*; *DE-Gal4*/*UAS-lacZ* / *y, w*; *DE-Gal4*/*UAS-PTEN^RNAi^*; (B,C,D) *y, w, eyFlp*/*y, w*; *FRT40w^+^ cl*/*FRT40iso* / *y, w, eyFlp*/*y, w*; *FRT40 w^+^ cl*/*FRT40 PTEN^117^*

**Figure 7.** (A) *y*, *w*, *eyFlp*/*y*, *w*; *FRT40 ubiGFP*/*FRT40iso* / *y*, *w*, *eyFlp*/*y*, *w*; *FRT40 ubiGFP*/*FRT40iso; ey-Gal4*/*UAS-Dilp-2* /  *y, w, hsFlp*/*y, w*; *FRT40 ubiGFP*/*FRT40 PTEN^117^* / *y, w, hsFlp*/*y, w*; *FRT40 ubiGFP*/*FRT40 PTEN^117^*; *ey-Gal4*/*UAS-Dilp-2;* (B) *y*, *w*, *eyFlp*/*y*, *w*; *FRT40 ubiGFP*/*FRT40iso* / *y*, *w*, *eyFlp*/*y*, *w*; *FRT40 ubiGFP*/*FRT40 TOR^2L19^* / *y*, *w*, *eyFlp*/*y*, *w*; *FRT40 ubiGFP*/*FRT40 TOR^2L19^*; *arm-Gal4*/*UAS-Imp-L2* / *y*, *w*, *eyFlp*/*y*, *w*; *FRT40 ubiGFP*/*FRT40 PTEN^117^* /  *y*, *w*, *eyFlp*/*y*, *w*; *FRT40 PTEN^117^, ubiGFP*/*FRT40 TOR^2L19^* / *y*, *w*, *eyFlp*/*y*, *w*; *FRT40 PTEN^117^, ubiGFP*/*FRT40 TOR^2L19^*; *arm-Gal4*/*UAS-Imp-L2*

**Figure 2 – figure supplement 1.** *y, w, hsFlp*/*y, w*; *FRT40 ubiGFP*/*FRT40iso* / *y, w, hsFlp*/*y, w*; *FRT40 ubiGFP*/*FRT40 PTEN^117^*

**Figure 2 – figure supplement 2.** *y, w, hsFlp*/*y, w*; *FRT40 ubiGFP*/*FRT40 PTEN^117^*

**Figure 2 – figure supplement 3.** *y, w, hsFlp*/*y, w*; *FRT40 w^+^*/*FRT40iso* / *y, w, hsFlp*/*y, w*; *FRT40 w^+^*/*FRT40 PTEN^117^*

**Figure 2 – figure supplement 4.** *y, w, hsFlp*/*y, w*; *FRT40 w^+^*/*FRT40iso* / *y, w, hsFlp*/*y, w*; *FRT40 w^+^*/*FRT40 PTEN^117^*

**Figure 2 – figure supplement 5.** *y, w, hsFlp*/*y, w*; *FRT40 w^+^*/*FRT40iso* / *y, w, hsFlp*/*y, w*; *FRT40 w^+^*/*FRT40 PTEN^117^*

**Figure 2 – figure supplement 6.** *y, w, eyFlp*/*y, w*; *FRT40 ubiGFP*/*FRT40iso* / *y, w, eyFlp*/*y, w*; *FRT40 ubiGFP*/*FRT40 PTEN^117^*

**Figure 3 – figure supplement 1.** *y, w, hsFlp, UAS-GFP*; *FRT40 tubGal80*/*FRT40iso*; *tubGal4*/+ / *y, w, hsFlp, UAS-GFP*; *FRT40 tubGal80*/*FRT40iso*; *tubGal4*/*UAS-p35* / *y, w, hsFlp*, *UAS-GFP*; *FRT40 tubGal80*/*FRT40 PTEN^117^*; *tubGal4*/+ / *y, w, hsFlp*, *UAS-GFP*; *FRT40 tubGal80*/*FRT40 PTEN^117^*; *tubGal4*/*UAS-p35*

**Figure 3 – figure supplement 2.** *y, w, hsFlp*/*y, w*; *FRT40 ubiGFP*/*FRT40 PTEN^117^*

**Figure 4 – figure supplement 1.** *y, w, eyFlp*/*y, w*; *FRT82 ubiGFP/FRT82iso* / *y, w, eyFlp*/*y, w*; *FRT40 PTEN^117^*; *FRT82 PTEN^genomic rescue^, ubiGFP*/*FRT82iso* / *y, w, eyFlp*/*y, w*; *FRT82 ubiGFP*/*FRT82 FoxO^Δ94^* / *y, w, eyFlp*/*y, w*; *FRT40 PTEN^117^*; *FRT82 PTEN^genomic rescue^, ubiGFP*/*FRT82* *FoxO^Δ94^* / *y, w, eyFlp*/*y, w*; *FRT82 ubiGFP*/*FRT82InR^5545^* / *y, w, eyFlp*/*y, w*; *FRT40 PTEN^117^*; *FRT82 PTEN^genomic rescue^*, *ubiGFP*/*FRT82 InR^5545^* / *y, w, eyFlp*/*y, w*; *FRT82 ubiGFP*/*FRT82 Rheb^2G5^* / *y, w, eyFlp*/*y, w*; *FRT40 PTEN^117^*; *FRT82 PTEN^genomic rescue^, ubiGFP*/*FRT82 Rheb^2G5^* / *y, w, eyFlp*/*y, w*; *FRT82 ubiGFP/FRT82 PI3K92E^2H1^* / *y, w, eyFlp/y, w*; *FRT40 PTEN^117^*; *FRT82 PTEN^genomic rescue^, ubiGFP*/*FRT82 PI3K92E^2H1^*

**Figure 4 – figure supplement 2.** *y, w, eyFlp*/*y, w*; *FRT40 cl w^+^*/*FRT40iso* / *y, w, eyFlp*/*y, w*; *FRT40 cl w^+^*/*FRT40 PTEN^117^*

**Figure 4 – figure supplement 3.** *y, w*; *GMR-Gal4*/+; *UAS-lacZ*/+ / *y, w*; *GMR-Gal4*/*UAS-PTEN^RNAi^* / *y, w*; *GMR-Gal4*, *Thor1*/+; *EP(dFoxO)*/+ / *y, w*; *GMR-Gal4*, *Thor1*/*UAS-PTEN^RNAi^*, *EP(dFoxO)*/+

**Figure 4 – figure supplement 4.** *y, w, hsFlp, UAS-myrGFP*/*y, w*; *FRT40 tub-Gal80*/ *FRT40iso*; *tub-Gal4*/+ / *y, w, hsFlp*, *UAS-myrGFP*/*y, w*; *FRT40 tub-Gal80*/*FRT40 PTEN^117^*; *tub-Gal4*/+ / *y, w, hsFlp*, *UAS-myrGFP*/*y, w*; *FRT40 tub-Gal80*/*FRT40iso*; *tub-Gal4*/*EP(dFoxO)* / *y, w, hsFlp, UAS-myrGFP*/*y, w*; *FRT40 tub-Gal80*/*FRT40 PTEN^117^*; *tub-Gal4*/*EP(dFoxO)*

**Figure 4 – figure supplement 5.** *y, w, hsFlp, UAS-myrGFP*/*y, w*; *FRT40 tub-Gal80*/*FRT40 PTEN^117^*; *tub-Gal4*/+ / *y, w, hsFlp, UAS-myrGFP*/*y, w*; *FRT40 tub-Gal80*/*FRT40 PTEN^117^*; *tub-Gal4*/*UAS-Tsc1 UAS-Tsc2* / *y, w, hsFlp, UAS-myrGFP*/*y, w*; *FRT40 tub-Gal80*/*FRT40iso*; *tub-Gal4*/*UAS-Tsc1 UAS-Tsc2*

**Figure 4 – figure supplement 6.** *y, w, eyFlp*/*y, w; FRT40 tub-Gal80*/*FRT40iso; tub-Gal4*/+ / *y, w, eyFlp*/*y, w; FRT40 tub-Gal80*/*FRT40iso; tub-Gal4*/*UAS-Tsc1 UAS-Tsc2* / *y, w, eyFlp*/*y, w*; *FRT40 tub-Gal80*/*FRT40 PTEN^117^*; *tub-Gal4*/+ / *y, w, eyFlp*/*y, w*; *FRT40 tub-Gal80*/*FRT40 PTEN^117^*; *tub-Gal4*/*UAS-Tsc1 UAS-Tsc2*

**Figure 4 – figure supplement 7.** *y*, *w, eyFlp*/*y*, *w*; *FRT40 cl w^+^*/*FRT40iso* / *y*, *w, eyFlp*/*y*, *w*; *FRT40 cl w^+^*/*FRT40 TOR^EP2353^* / *y*, *w, eyFlp*/*y*, *w*; *FRT40 cl w^+^*/*FRT40 PTEN^117^* / *y*, *w, eyFlp*/*y*, *w*; *FRT40 cl w^+^*/*FRT40 PTEN^117^*, *TOR^EP2353^*

**Figure 5 – figure supplement 1**. *y, w,* *hsFlp, UAS-myrGFP*/+; *FRT40 PTEN^117^*/*FRT40 tubGal80*; *tubGal4*/+ / *y, w,* *hsFlp, UAS-myrGFP*/+; *FRT40 PTEN^117^*/*FRT40 tubGal80*; *tubGal4*/*UAS*-*Slif^anti^* / *y, w,* *hsFlp, UAS-myrGFP*/*UAS-Atg5^RNAi^*; *FRT40 PTEN^117^*/*FRT40 tubGal80*; *tubGal4*/+ / *y, w,* *hsFlp, UAS-myrGFP*/*UAS-Atg5^RNAi^*; *FRT40 PTEN^117^*/*FRT40 tubGal80*; *tubGal4*/*UAS-Slif^anti^* / *y, w*, *hsFlp, UAS-myrGFP*/+; *FRT40 PTEN^117^*/*FRT40 tubGal80*; *tubGal4*/*UAS-p35* / *y, w,* *hsFlp, UAS-myrGFP*/+; *FRT40 PTEN^117^*/*FRT40 tubGal80*; *tubGal4*/*UAS-p35*, *UAS*-*Slif^anti^*

**Figure 5 – figure supplement 2**. *y, w,* *hsFlp, UAS-myrGFP*/+; *FRT40 PTEN^117^*/*FRT40 tubGal80*; *tubGal4*/*UAS-p35*, *UAS*-*Slif^anti^*

**Figure 5 – figure supplement 3**. *y, w,* *hsFlp, UAS-myrGFP*/+; *FRT40 PTEN^117^*/*FRT40 tubGal80*; *tubGal4*/+ / *y, w,* *hsFlp, UAS-myrGFP*/+; *FRT40 PTEN^117^*/*FRT40 tubGal80*; *tubGal4*/*UAS*-*Slif^anti^* / *y, w,* *hsFlp, UAS-myrGFP*/*UAS-Atg5^RNAi^*; *FRT40 PTEN^117^*/*FRT40 tubGal80*; *tubGal4*/+ / *y, w,* *hsFlp, UAS-myrGFP*/*UAS-Atg5^RNAi^*; *FRT40 PTEN^117^*/*FRT40 tubGal80*; *tubGal4*/*UAS-Slif^anti^* / *y, w,* *hsFlp, UAS-myrGFP*/+; *FRT40 PTEN^117^*/*FRT40 tubGal80*; *tubGal4*/*UAS-p35*, *UAS*-*Slif^anti^*

**Figure 6 – figure supplement 1.** *y, w, hsFlp*/*y, w*; *FRT40 ubiGFP*/*FRT40PTEN^117^* / *y, w, hsFlp*/*y, w*; *FRT40 ubiGFP*/*FRT40iso*

**Figure 7 – figure supplement 1.** *y, w, hsFlp*/*y, w*; *FRT40 ubiGFP*/*FRT40 PTEN^117^*; *FRT82 armLacZ*/*FRT82iso* / *y, w, hsFlp*/*y, w*; *FRT40 ubiGFP*/*FRT40 PTEN^117^*; *FRT82 armLacZ*/*FRT82 Tsc1^Q87X^*

**Figure 7 – figure supplement 2.** *y, w, eyFlp*/*y, w*; *FRT40 ubiGFP*/*FRT40iso* / *y, w, eyFlp*/*y, w*; *FRT40 ubiGFP*/*FRT40 TOR^2L19^* / *y, w, eyFlp*/*y, w*; *FRT40iso*/*FRT40 PTEN^117^, ubiGFP* / *y, w, eyFlp*/*y, w*; *FRT40 PTEN^117^, ubiGFP*/*FRT40 TOR^2L19^*

**Figure 7 – figure supplement 3.** *y, w, eyFlp*/*y, w*; *FRT40iso*/*FRT40 PTEN^117^, ubiGFP* / *y, w, eyFlp*/*y, w*; *FRT40 PTEN^117^, ubiGFP*/*FRT40 TOR^2L19^*

**Figure 7 – figure supplement 4.** *y, w, hsFlp*/*y, w*; *FRT40 ubiGFP*/*FRT40iso* / *y, w, hsFlp*/*y, w*; *FRT40 TOR^2L19^*/*FRT40 ubiGFP* / *y, w, hsFlp*/*y, w*; *FRT40 TOR^2L1^*/*FRT40 ubiGFP* / *y, w, hsFlp*/*y, w*; *FRT82 PKB^3^*/*FRT82 ubiGFP*

**Figure 7 – figure supplement 5.** *y*, *w, eyFlp*/*y*, *w*; *FRT40 ubiGFP*/*FRT40iso* / *y*, *w, eyFlp*/*y*, *w*; *FRT40 ubiGFP*/*FRT40 TOR^2L19^* / *y*, *w, eyFlp*/*y*, *w*; *FRT40 ubiGFP*/*FRT40 PTEN^117^* / *y*, *w*, *eyFlp*/*y*, *w*; *FRT40 PTEN^117^, ubiGFP*/*FRT40 TOR^2L19^*

**Figure 7 – figure supplement 6.** *y*, *w*, *eyFlp*/*y*, *w*; *FRT40 PTEN^117^, ubiGFP*/*FRT40 TOR^2L19^* / *y*, *w*, *eyFlp*/*y*, *w*; *FRT40 PTEN^117^, ubiGFP*/*FRT40 TOR^2L19^*; *arm-Gal4*/*UAS-Imp-L2*
